# Carpet Beetle Species (Coleoptera: Dermestidae) in Austrian Heritage Interiors and Their European Distributions

**DOI:** 10.3390/insects17060654

**Published:** 2026-06-22

**Authors:** Peter Brimblecombe, Graham Holloway, Pascal Querner

**Affiliations:** 1 Department of Marine Environment and Engineering, National Sun Yat-Sen University, Kaohsiung 80424, Taiwan; 2 School of Environmental Sciences, University of East Anglia, Norwich NR4 7TJ, UK; 3 Cole Museum of Zoology, HLS Building, University of Reading, Reading RG6 6EX, UK; g.holloway@reading.ac.uk; 4 Department of Integrative Biology and Biodiversity Research, Institute of Zoology, University of Natural Resources and Life Sciences, Gregor-Mendel-Straße 33, 1180 Vienna, Austria; pascal.querner@boku.ac.at; 5 Institute of Conservation, University of Applied Arts Vienna, Salzgries 14/3, Stock, 1010 Vienna, Austria

**Keywords:** Dermestidae, Austria, Central and Western Europe, integrated pest management, insect traps, catch rate, libraries, museums, storerooms

## Abstract

Museum collections are at risk from insects. A changing climate or increased amounts of imported materials, exhibition loans and international travel, can increase exotic species. Heritage properties are often monitored for pests, so we used trapping data from 31 Austrian museums, libraries and storerooms. The carpet beetles *Anthrenus* spp. and *Attagenus* spp. studied here, showed that the catch of these two species in buildings was correlated. Unheated libraries show high catch rates for *Anthrenus* spp., *Attagenus* spp. seemed more often caught in heated/urban museums. *Anthrenus verbasci*, *Anthrenus olgae* and *Anthrenus museorum* account for almost 98% of our catch. *Anthrenus verbasci* and *Anthrenus olgae* are commonly found occurring together suggesting they form a core ecological pair, found in most buildings. Rarer *Anthrenus fuscus* appears typically at locations lacking winter heating. *Attagenus smirnovi* and *Attagenus unicolor* accounted for 95% of this genus in the buildings. There are notable differences in the types of carpet beetle across European heritage environments. *Anthrenus olgae*, often trapped in Austria, is uncommon elsewhere, while *Anthrenus sarnicus*, fairly common in the UK, is rare elsewhere. Not enough is known about the range of heritage insects across Europe, which is increasingly relevant to management under a changing climate.

## Simple Summary

Museums are concerned about damage to collections by insects; a risk that can increase under a changing climate or through larger numbers of invasive pests introduced with imported materials, exhibition loans and international travel. These changes have led to the appearance of novel species of silverfish and beetles in these buildings. Carpet beetles are dermestids (2000 species worldwide). Two important genera are studied here *Anthrenus* and *Attagenus*, which were caught in 31 museums, libraries and storerooms from locations across the east of Austria. The catch of these two types of carpet beetles in the buildings was loosely proportionate. However, colder buildings seemed to favour species such as *Anthrenus fuscus*. *Anthrenus olgae*, though frequently caught in Austria, appears less common elsewhere, suggesting carpet beetles have different ranges in Europe. A warmer future is likely to mean an increasing number of different species becoming a potential threat to heritage collections.

## 1. Introduction

Carpet beetles are common pests indoors and are found in all continents except Antarctica. Their destructive effects are in evidence from Ancient Egyptian times [1] and they proved troublesome historically in Europe [2]. The pest remains common, so is frequently found in museums [3,4], archives [5], libraries [6,7], historic houses [8] and museum storerooms [9], where they are of concern because they damage, wool furnishings and carpets, clothing, natural history specimens etc [10,11]. These dermestid beetles are typically members of the genera: *Anthrenus* and *Attagenus* with a large number of species considered as pests [12].

The late 20th century saw increasing interest in Integrated Pest Management of the heritage environment [4,13]. As part of preventive conservation [14], it is an evidence-based approach to controlling pests that threaten collections, rather than relying on routine chemical treatments. It relies on monitoring and identification with regular inspection using sticky traps, identification of pest species and detecting sites of infestation and longer-term trends. Prevention focuses on the control of food sources (cleaning), access routes and maintaining appropriate environmental conditions. The aim is to be minimally invasive, with an emphasis on non-chemical control methods such as attentive housekeeping, freezing infested objects and anoxic treatments or where needed minimal and targeted use of pesticides. While the approach balances collection preservation, human safety and environmental responsibility, it requires input from entomology, building science and heritage science.

Recent times have seen a widening range of insect pests in museums, possibly as a result of increased international movement of goods, more frequent travel and a rising exchange of exhibitions. Notable has been the increased presence of previously uncommon silverfish such as *Ctenolepisma longicaudatum* Escherich, 1905 [15] and *C. calvum* Ritter, 1910 [16] along with increased observations of the skin beetle, *Reesa vespulae* Milliron, 1939 [17].

Carpet beetles originate in nature in bird, mammal or *Hymenoptera* nests where they feed on feathers, hair, skin along with small or dead vertebrates and insects. They typically prefer temperatures between 18–29 °C as warmer conditions shorten their life cycle. Moderate humidity 50–70% is preferred, especially during the larval stage. Some species, along with food pests, are well adapted to human homes and live synanthropically. Some species like varied carpet beetle *Anthrenus verbasci* (Linnaeus 1767) have a worldwide distribution, others are found in smaller regions or only outdoors. Almost 2000 species of dermestids have been recognised [18], with the most important genera *Anthrenus*, *Attagenus* and *Dermestes*. In addition to the common genera: other dermestids found in museums include: *Thylodrias contractus* Motschulsky, 1839 (odd beetle) [19], *Reesa vespulae* (skin or American wasp beetle) [17] *Trogoderma glabrum* Herbst, 1783 (glabrous cabinet beetle) [20,21], *Trogoderma angustum* Solier, 1849 (Berlin beetle) and *Trogoderma versicolor* Creutzer, 1799. Although these were occasionally found on traps set out in Austrian museums, these were just small catches [22].

Museum and urban pest ecology is the study of how insects and other organisms live, reproduce, and interact with collections and building environments. These represent a resource-limited indoor ecosystem, where unlike natural ecosystems, museum pest ecology is shaped by the provision of human resources and strong control by environmental gradients rather than interspecies interactions, thus aligning with Henry Gleason’s individualistic or continuum theory [22,23]. Museum pest communities typically have low species diversity with a few apparently adapted species dominant, they seem to persist well in the indoor environment (but maybe rather poorly compared with their performance under wild conditions). Museum and other types of buildings can resemble “islands” that can be isolated within the urban fabric (concrete or asphalt) and perhaps with their ecology shaped by a kind of island biogeography. Here isolation (distance to another building or large building—as mainland), habitat size, immigration (by human introduction) and possible extinction (with efficient IPM activities), along with clear boundaries to the outdoors, are likely to all influence its local indoor diversity [22]. Nevertheless, the surrounding environment can affect the indoor catch of carpet beetles e.g., *Anthrenus museorum* (Linnaeus 1761) may be a more common insect in rural settings, while *Anthrenus verbasci* seems more urban [24]. Whether natural habitats like parks, forests or gardens are a natural source of *Anthrenus* species in buildings is often discussed but seldom proven.

The research presented here aims to compare the catch of a variety of carpet beetles from a range of Austrian heritage environments and compare the results with the species distribution across Europe. We wish to compare the geographic ranges of the better-known species in heritage buildings and compare this with other databases. This can help to monitor the spread of new species and evaluate increasing risk of damage to collections.

## 2. Material and Methods

**Figure 1:**
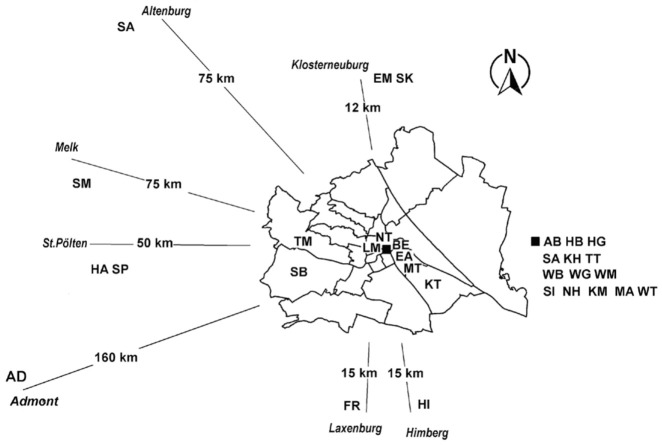
Building locations in the districts of Vienna (outlined) and Lower Austria, with Admont further away in the state of Styria. The locations beyond Vienna are not to scale, though are shown as the direction from the center of Vienna along with the distances from Innere Stadt (central district of Vienna marked by a black square). The two-letter location codes are listed in Table 1. Sites that are in Innere Stadt are listed to the right of the other Vienna districts.

This study used insect trapping data from museums, libraries and storerooms from locations across the east of Austria (Figure 1). These are compared with online databases and expert evaluations of museum insects from European countries such as Germany, Switzerland, France, Italy, Spain, Czech Republic and the UK [25,26,27]. Abundant species were additionally checked in the large general database Global Biodiversity Information Facility [28] for their European-wide distribution.

### 2.1. Study Locations

Insect traps in the current study were set out in buildings chosen to cover a range of building uses and interior climates. These were: (i) libraries, typically unheated monastic libraries in historic buildings, often in rural areas with few visitors, (ii) museum galleries that were open to the public (some heated, but others with HVAC systems) and (iii) storerooms for museum collections and archives, which try to maintain stable interior climates and are typically closed to casual visitors. The buildings are listed along with insect trapping data in Table 1. In larger buildings, libraries, exhibition rooms and storage rooms, it was possible to extract the catch for separate areas.

**Table 1:** Buildings and dermestid catch that form part of the current study. The **Code** is two letters followed by a designator: libraries l, museums m and storage s. **Control** describes the type of climate control within the building HVAC heating ventilation and air conditioning, core: cooling through concrete core activation in the floor and ceiling, mobile de-humidifiers or humidifiers. **Years** is the duration of trapping data available. ***Anthrenus* spp.** and ***Attagenus* spp.** listing firstly the total numbers of adults and larvae caught combined followed by the numbers of adults of individual species: *Anthrenus verbasci*, *Anthrenus olgae*, *Anthrenus museorum*, *Anthrenus scrophulariae* and *Anthrenus fuscus*, and *Attagenus smirnovi*, *Attagenus unicolor* and *Attagenus pellio*. The number of larvae caught on traps are not listed. The final row gives the sums from all buildings. High catches (>500 ind.) are marked in bold.

Code	Building	Setting	Control	Traps	Years	*Anthrenus* spp.	*Attagenus* spp.
ABs	Albertina Storage	urban	HVAC	15	4	8, 0, 3, 0, 0, 0	1, 0, 0, 0
ADl	Admont Abbey	rural	none	100	3	**977**, 0, 0, 88, 1, 0	12, 0, 0, 12
BAm	Basteihalle	urban	HVAC	22	5	14, 10, 0, 0, 0, 0	20, 18, 0, 0
BEm	Oberes Belvedere Museum	urban	HVAC	75	5	203, 18, 18, 0, 1, 0	148, 45, 8, 0
EAs	Exilarte Zentrum Storage	urban	HVAC	17	5	7, 1, 2, 0, 0, 0	24, 8, 0, 0
EMs	Klosterneuburg Storage	rural	HVAC	77	5	60, 34, 5, 0, 0, 0	15, 0, 13, 0
FRm	Franzensburg Castle M.	rural	none	130	5	315, 1, 1, 169, 5, 4	173, 0, 14, 97
HAs	Hart Storage	rural	none	85	5	94, 18, 16, 0, 0, 0	2, 0, 0, 1
HBs	Archive and Studio	urban	heating	60	2	62, 10, 0, 5, 0, 25	**920**, 453, 0, 0
HGs	Storage	rural	heating	137	5	452, 4, 136, 0, 8, 0	172, 101, 2, 0
HIs	Storage	rural	core	588	5	155, 64, 24, 0, 3, 0	56, 0, 21, 0
HMm	Heeresgeschichtliches M.	urban	heating	47	5	216, 4, 86, 0, 0, 0	101, 76, 0, 0
KBl	Capuchin Library	urban	none	21	5	63, 2, 18, 0, 0, 0	11, 0, 0, 0
KHm	Large Museum	urban	heating	710	3	**1005**, 135, 219, 0, 2, 0	**1295**, 432, 0, 0
KTs	Belvedere Storage	rural	core	56	5	5, 1, 1, 0, 0, 0	3, 0, 1, 0
LMs	Leopold Storage	urban	HVAC	56	5	21, 0, 7, 0, 0, 0	0, 0, 0, 0
MAs	MAK Storage	urban	none	60	5	101, 8, 11, 1, 0, 0	41, 1, 8, 0
NHlms	Natural History Museum	urban	heating	825	5	**773**, 59, 188, 3, 0, 0	**1663**, **632**, 116, 0
NTs	Narrenturm Museum	urban	none	43	3	153, 29, 29, 0, 0, 6	30, 0, 7, 0
SAl	Altenburg Abbey Library	rural	none	32	5	112, 0, 0, 64, 1, 1	29, 1, 0, 28
SBms	Schoenbrunn Museum	urban	none	236	5	**1058**, 117, 390, 2, 0, 0	336, 1, 168, 0
SIm	Sisi Museum	urban	none	100	5	282, 31, 90, 0, 3, 0	383, 19, 180, 0
SKl	Klosterneuburg Library	rural	none	43	5	325, 12, 91, 1, 0, 1	82, 1, 61, 0
SMl	Melk Library	rural	none	113	5	474, 22, 140, 17, 0, 0	24, 0, 3, 1
SPs	Kulturdepot Storage	rural	core	191	5	71, 16, 14, 3, 1, 0	10, 0, 0, 5
TMms	Archive and Museum	urban	heat	245	5	141, 21, 17, 0, 2, 0	141, 12, 15, 0
TTlms	Specialist Museum	urban	HVAC	69	3	262, 23, 107, 0, 0, 0	143, 55, 0, 0
WBls	Storage	urban	none	172	5	151, 8, 23, 0, 0, 0	54, 0, 21, 0
WHs	Storage	rural	core	367	5	50, 15, 1, 2, 1, 0	6, 0, 2, 0
WMm	Museum	urban	HVAC	122	3	102, 8, 12, 0, 1, 0	**579**, 244, 2, 0
WTs	Storage	urban	heating	186	3	134, 11, 13, 0, 1, 0	62, 13, 0, 0
SUM						7846, 682, 1662, 355, 30, 37	6536, 2112, 642, 144

### 2.2. Insect Trapping

Carpet beetles were trapped, along with other arthropods, using traps (PPS GmbH, Robert-Bosch-Straße 6, 73278 Schlierbach, Germany), either sticky blunder traps (Catchmaster, Bayonne, NJ, USA) or a smaller number of Finicon pheromone traps. The latter were set out to attract the webbing clothes moth, *Tineola bisselliella* Hummel, 1823, but other insects were caught as they blundered in [29]. The traps are made of cardboard with pressure-sensitive glue and are typically placed at regular intervals, every 10 m or so along the walls. They were replaced when full or in the spring and autumn of every year and were set out as part of Integrated Pest Management activities, and more recently a project on modelling museum pests. The contents of the traps were examined three to five times per year to determine insects present. Identification of the insects caught on traps utilised different keys [30,31,32] and was largely based on morphology. The insect trapping data used in this paper is available at Zenodo [33]. The buildings studied vary widely in size, so the number of traps set out varied, which means there are differences in the trapping effort between buildings. Thus, any comparison needs to account for the number of traps and the length of the trapping observations. Here the trapping effort was expressed as a catch rate, in which the catch was divided by the number of traps and the number of years over which the traps were placed out. Thus, the annual catch rate was catch per trap-year in the building. This means only a weak dependence on building size, even though the number of traps set out is dependent on building size and function.

### 2.3. Taxonomy

In this paper we have followed the increasingly recommended practice of writing all taxonomic levels in italic script [34] and as both genera begin with the letter-A we have avoided abbreviation [35]. The family *Dermestidae* (Latreille, 1804) contains almost 2000 species [18], which results in a complex nomenclature. The insects trapped were identified using some important keys [36,37,38].

### 2.4. Data Analysis and Statistics

The integer nature of the trapping data and the likelihood of skewed distributions (i.e., with many zeroes), means it typically fails the Shapiro-Wilk test [36] for normality. Thus, we have used non-parametric statistics and expressed central tendency and dispersion as the median and lower (*Q*_1_) and upper quartiles (*Q*_3_) rather than mean and standard deviation. Box-and-whisker plots have been used to display the range within data sets. Here the boxes represent the *Q*_1_ and *Q*_3_, with the median denoted by the line across the box, and a cross to represent the mean. The whiskers show the range of all other points, except those that are deemed as outliers (i.e., values over 1.5 times the interquartile range beyond *Q*_1_ or *Q*_3_. The significance of differences between means of catch and catch used the Mann-Whitney and Wilcoxon signed-rank tests, the latter for correlated data. The Kruskal-Wallis test was used to determine the significance of differences between multiple data sets, subsequently applying Dunn’s test to assess pairwise comparisons between groups. When correlating catches, we used Kendall *τ* as an effective measure of the association between two ranked variables [37], with *τ* resembling the familiar parametric correlation coefficient *r*. It is less sensitive to outliers than Pearson’s correlation and robust at small sample sizes. The Jaccard similarity index [38], *J* has been used to represent overlap between species in the buildings: *J* = *B*_11_/(*B*_01_ + *B*_10_ + *B*_11_), where *B*_11_ represents the number of buildings where both species were present and *B*_01_ and *B*_10_ buildings where only one or the other was present.

## 3. Results and Discussion

The carpet beetles (*Dermestidae*) found included *Anthrenus verbasci* (varied carpet beetle), *Anthrenus olgae* Kalík, 1946, *Anthrenus museorum* Linnaeus, 1761 [39] (museum beetle), *Anthrenus scrophulariae* Linnaeus, 1758 (common carpet beetle), *Anthrenus fuscus* Olivier, 1789, *Attagenus smirnovi* Zhantiev, 1973 (brown carpet beetle or vodka beetle) [40,41], *Attagenus unicolor* Brahm, 1971 (black carpet beetle), *Attagenus brunneus* Faldermann, 1835 and *Attagenus pellio* Linnaeus, 1758 (two spotted carpet beetle). The larvae were not recorded at the species level as it can be difficult to distinguish them on sticky traps. Recent re-descriptions and differentiation of species is detailed with references at various point in the text (mostly from one of the current authors: Holloway).

### 3.1. Overall Catch from Buildings

**Figure 2:**
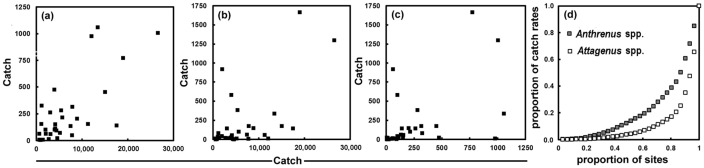
(**a**) The catch of *Anthrenus* spp. as a function of total arthropod catch in the buildings. (**b**) Catch of *Attagenus* spp. as a function of total arthropod catch. (**c**) Catch of *Attagenus* spp. as a function of *Anthrenus* spp. catch. (**d**) Lorenz plot of the cumulative proportion of the catch rates as a function of the proportion of the number of buildings, with *Anthrenus* spp. as grey squares and *Attagenus* spp. as open squares.

Figure 2a shows the catch of *Anthrenus* spp. as a function of total arthropod catch in the buildings. The Kendall rank correlation coefficient *τ* of 0.45 reveals a significant relationship (*p*_2_ < 0.001) between this catch and the general presence of arthropods in buildings. This was also true of *Attagenus* spp. (Figure 2b), although the relationship is somewhat weaker (*τ* = 0.32; *p*_2_~0.011). Figure 2c shows, perhaps unsurprisingly, that the catch of *Attagenus* spp. in buildings is correlated with *Anthrenus* spp. catch (*τ* = 0.45; *p*_2_ < 0.001). *Attagenus* spp. appear less evenly spread among the buildings than the *Anthrenus* spp. as might be perceived by the greater scatter in Figure 2b.

**Figure 3:**
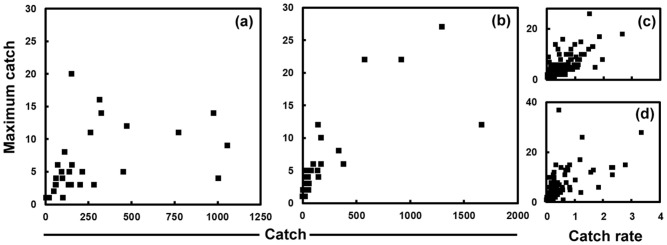
(**a**) Maximum catch of *Anthrenus* spp. observed for any trap within each building as a function of overall catch of *Anthrenus* spp. (**b**) Maximum annual catch of *Attagenus* spp. in a year as a function of its overall catch. (**c**) Similar plot for *Anthrenus* spp. but as a function of the annual catch rate. (**d**) Similar plot for *Attagenus* spp. as a function of the annual catch rate.

The evenness of the distribution of the genera among buildings is illustrated by the Lorenz plot of *Anthrenus* spp. and *Attagenus* spp. (Figure 2d). It shows the cumulative proportion of the catch rates of *Anthrenus* spp. and *Attagenus* spp. as a function of the proportion of the number of buildings. The sharper angled (more concave) curve for *Attagenus* spp. shows it to be more unevenly distributed. The uneven presence in the buildings means that it is possible for the overall catch rate to be a poor indicator of overall presence, because infestations can mean that the insects are found in high numbers, but at just a few trapping sites. However, there was a correlation (Figure 3a) between the maximum catch of *Anthrenus* spp. from any trapping site and the total catch (*τ* = 0.56; *p*_2_ < 0.001). Figure 3b shows a similar situation for *Attagenus* spp. *τ* = 0.78; *p*_2_ < 0.001). The maximum catch from each year is similarly well-correlated with the yearly catch rates for both *Anthrenus* spp. (Figure 3c) and *Attagenus* spp. (Figure 3d). These all confirm that maximum catch is related to the overall catch as has previously been noted in the case of silverfish (*Lepismatidae*) [42].

### 3.2. Building Use

**Figure 4:**
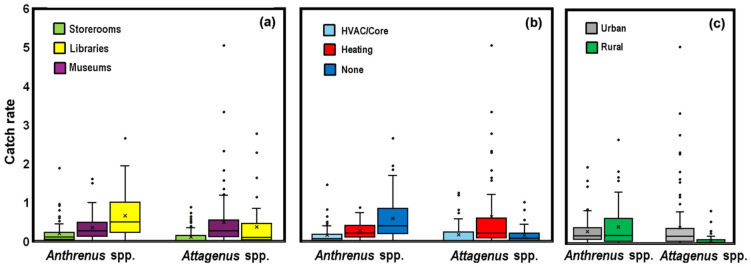
(**a**) Box-and-whisker plots of the yearly catch rates from storerooms, galleries of museums and libraries within the buildings, for *Anthrenus* spp. and *Attagenus* spp. (**b**) Box-and-whisker plots of the yearly catch rates of *Anthrenus* spp. and *Attagenus* spp. in buildings with different types of climate control. (**c**) Box-and-whisker plots of the yearly catch rates of *Anthrenus* spp. and *Attagenus* spp. in buildings with urban and rural locations. Note: symbols are explained in Section 2.4.

Figure 4 shows the catch rates each year for both *Anthrenus* spp. and *Attagenus* spp. in relation to the building function, climate control and location. Storerooms, museum spaces and libraries show different catch rates for *Anthrenus* spp. (Figure 4a, Kruskal Wallis *p* < 0.001), with median catch rates of 0.12, 0.28 and 0,5, respectively. The post-hoc Dunn’s test indicated all pairs are significantly different (*p* < 0.05). A similar test for *Attagenus* spp. also shows significant differences (*p* < 0.001; Dunn’s test all comparisons *p* < 0.01), with median catch rates of 0.026, 0.269 and 0.103, respectively. Storerooms appear to have the lowest catch for both genera. The libraries have the highest catch rates for *Anthrenus* spp. though it is the museums in the case of *Attagenus* spp.

A similar picture emerges for the type of climate control in the buildings. with the Kruskal-Wallis test suggesting significant differences for both *Anthrenus* spp. and *Attagenus* spp. (*p* < 0.001). Buildings with HVAC, heating and no climate control show different catch rates for *Anthrenus* spp. (Figure 4b, Kruskal Wallis *p* < 0.0025), with median catch rates of 0.078, 0.222 and 0.408, respectively. The median catch rates for *Attagenus* spp. caught in rooms were 0.026, 0.215 and 0.085, although the pairwise differences are not significant (*p* > 0.1) between HVAC and unheated spaces. The similarity between the patterns for the type of use of the building and climate control, means that it is difficult to distinguish which of these two factors might control the different catch rates. There were too few buildings to attempt a two-way analysis.

The median catch rate for *Anthrenus* spp. in urban and rural buildings, 0.19 and 0.20 are not significantly different (Mann-Whitney *p*_2_ > 0.8). In the case of *Attagenus* spp. the median catch rates of 0.19 and 0.026 were significantly different (*p* < 0.001), with the insects showing much lower catch rates in rural locations, perhaps part of the rural-urban differences mentioned earlier [24].

### 3.3. Individual Species

**Figure 5:**
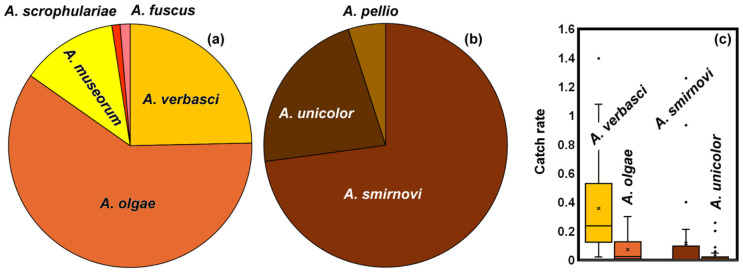
(**a**) Pie diagrams for the catch of various (**a**) *Anthrenus* spp. and (**b**) *Attagenus* spp. (**c**) Box-and-whisker plots of the yearly catch rates of *Anthrenus verbasci*, *Anthrenus olgae*, *Attagenus smirnovi* and *Attagenus unicolor*. Note: symbols in Figure 5c are explained in Section 2.4.

The total catch of *Anthrenus* spp. was 7846, but over 5000 were in larval form so only a third could be determined to species level as: *Anthrenus verbasci*, *Anthrenus olgae*, *Anthrenus museorum*, *Anthrenus scrophulariae* and *Anthrenus fuscus* (Figure 5a; Table 1), along with a single example of *Anthrenus querneri* Holloway 2024 [43]. The catch of *Attagenus* spp. was approximately 6500, with about 3500 in larval form, meaning that less than half could be determined to species level as *Attagenus smirnovi*, *Attagenus unicolor* and *Attagenus pellio* (Figure 5b; Table 1). Box-and-whisker plots of the yearly catch rates of *Anthrenus verbasci*, *Anthrenus olgae*, *Attagenus smirnovi* and *Attagenus unicolor* are shown in Figure 5c. A Wilcoxon signed-rank test indicated that there is a significant difference (*p* < 0.01) between the catch rates of both the two *Anthrenus* spp., but no significant difference (*p* < 0.1) for *Attagenus* spp.

*Anthrenus verbasci*, *Anthrenus olgae* and *Anthrenus museorum* account for almost 98% of the catch, with only 30 and 37 individuals as *Anthrenus scrophulariae* and *Anthrenus fuscus* (see Table 1), although it is possible that some larvae were also representative of these two species. The two most common species, *Anthrenus verbasci* and *Anthrenus olgae* were present in traps from at least 27 of the 31 buildings. The Jaccard similarity between *Anthrenus verbasci* and *Anthrenus olgae* is very high at ~0.82, so they are commonly found occurring together and appear to form a core ecological pair, so might have overlapping ecological requirements. As richness increases, *Anthrenus museorum* and *Anthrenus scrophulariae* are found (at 11 and 13 locations), although *Anthrenus scrophulariae* is caught some five times less frequently. There is occasional overlap between the *Anthrenus museorum* and *Anthrenus scrophulariae,* i.e., occur in the same buildings, but there are also cases where the buildings have only one of these species. Elevated catch rates of *Anthrenus museorum* were found at SA, FR and NH, while *Anthrenus scrophulariae* are caught at higher rates at HG, FR, SA and SI. The rarer *Anthrenus fuscus* appears only at five species-rich locations (FR, SA, SK, NT, HB) which typically have little winter heating, suggesting it may be a species that requires a cold winter period, perhaps to stimulate emergence from pupa as the temperature increases in the spring.

*Attagenus smirnovi* and *Attagenus unicolor* accounted for 95% of the *Attagenus* spp. identified, they are common and widespread. *Attagenus pellio* is much less frequent, it occurs in less than a quarter of the buildings. While *Attagenu pellio* sometimes co-occurs with the other two species, it is never found with both simultaneously. *Attagenus smirnovi* is particularly abundant at HB and in the library at NH. *Attagenus pellio* was notable at FR and SA, both rural buildings without substantial winter heating. These species are often found in locations with large numbers of dead insects such as cluster flies, *Pollenia* spp. (FR, NH) or with a food source (dust and dead insects) to be found underneath historic wooden floors. They can also feed on human hair, so can be abundant in areas with high visitor numbers (HB, NH). Dead Rodents or bird nests could also be a potential food source but were not found in the locations studied.

### 3.4. Overall Distribution Across Europe

**Figure 6:**
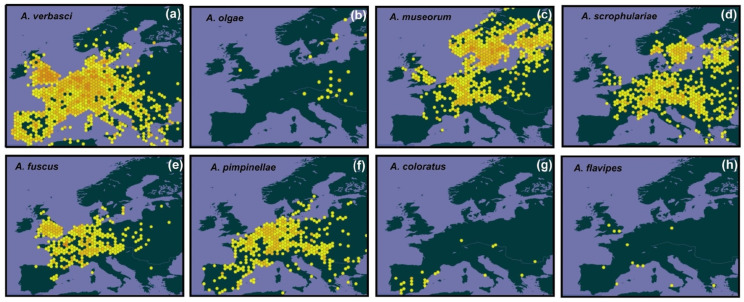
Distribution of reports of various *Anthrenus* spp. in Central and Western Europe from the GBIF data. (**a**) *Anthrenus verbasci*, (**b**) *Anthrenus olgae*, (**c**) *Anthrenus museorum*, (**d**) *Anthrenus scrophulariae*, (**e**) *Anthrenus fuscus*, (**f**) *Anthrenus pimpinellae*, (**g**) *Anthrenus coloratus* and (**h**) *Anthrenus flavipes*. Note: increasingly orange shades denote larger numbers.

Herrmann identifies 52 *Anthrenus* spp. and 50 *Attagenus* spp. from Europe [44]. The GBIF data was used to map various carpet beetles largely from natural and urban habitats across the European continent. Such maps give a sense of the potential for the insects to be transferred indoors and the dominance of interest in these species in Central and Western Europe. However, it is important to remember that these maps are based on reports, which can reflect interest or collection rather than the absolute abundance and incorporate misidentification (they also incorporate possible misidentification, for example no evidence that *A. pimpinellae* (Fabricius, 1775) occurs in Spain and no evidence that *A. scrophulariae* and *A. flavipes* occur and can persist in the UK). Nevertheless, the maps suggest an uneven distribution of these *Dermestidae* across Europe. Although *Anthrenus verbasci* is widely recorded, many other species are less commonly found. It is notable that *Anthrenus olgae*, which is frequently caught in Austrian heritage environments, is not at all common in the map of Figure 6b [45], where we see it is reported from Eastern Europe and Baltic countries. It was found previously as light infestations in the UK between 1984 and 1987, but its presence was not sustained [39]. *Anthrenus olgae* is quite small and might therefore be underrepresented in the GBIF database. It is also found in heritage buildings in the Czech Republic (Anna P. Email communication in 20 April 2026) and described as a common pest in Hungarian pest control [46].

The most abundant species in Europe is *Anthrenus verbasci* (Figure 6a), with just over 17,000 records showing in the GBIF database [47]. A third of these are from the UK, with Germany and France combining to form another third. It is quite ubiquitous with the beetle recorded in some thirty countries across Europe.

The second most abundant species is *Anthrenus museorum* (Figure 6c) that has just over 7000 European records in the GBIF database [48]. It seems most prevalent in Northwestern Europe, being very frequently recorded from Sweden, Norway and Finland accounting for two-thirds of the records.

*Anthrenus scrophulariae* (Figure 6d) is rather widely spread with about 10% of the European record in each of the five countries Sweden, Germany, Switzerland, Poland and Russia [49]. Notably the three species *Anthrenus scrophulariae*, *Anthrenus olgae* and *Anthrenus museorum* go unrecorded on the Iberian Peninsula.

Southern and Western Europe sees both *Anthrenus fuscus* (Figure 6e) and *Anthrenus pimpinellae* (Figure 6f) well recorded [50,51], and they appear across Central and Western Europe and the Mediterranean. *Anthrenus fuscus* is most frequently recorded in France, where a third are found and a quarter of the European records come from the UK. The GBIF database reveals that more than half the records of *Anthrenus pimpinellae* come from France, Germany and Switzerland. However, *Anthrenus pimpinellae* may in reality be up to 20 different species and Holloway found no evidence for self-sustaining populations in the British Isles [42]. *Anthrenus coloratus* (Figure 6g) [52] and *Anthrenus flavipes* (Figure 6h) are not often recorded, but do appear less likely to be present in Northern Europe.

*Anthrenus flavipes* (LeConte 1854) is regularly found in one building in Vienna (unpublished data) in large numbers. The large building is used for storing museum objects, but also food and other materials as part of a tax-free warehouse. The Guernsey carpet beetle *Anthrenus sarnicus* Mroczkowski, 1963 is typically limited to the British Isles, introduced to the UK [41] with the first record in South Kensington in 1963 [53]. However, these beetles have been found in one museum storage area in Vienna. *Anthrenus angustefasciatus* Ganglbauer, 1904 (a member of the *Anthrenus pimpinellae* complex) is relatively new to the British Isles, but as yet is not seen in museums, but it may be more widely found in north and western Europe [54,55]. *Anthrenus caucasicus* Reitter, 1881 was originally from Iran and Turkmenistan, but has been introduced to Austria, Latvia, Poland, and Slovakia in recent years [44]. *Anthrenus flavidus* Solskij, 1876, is known from Central Asia and China [56], but sometimes found in Europe [44].

**Figure 7:**
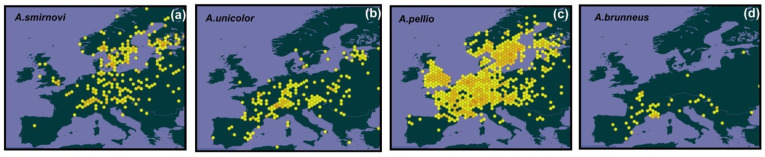
Distribution of reports of various *Attagenus* spp. in Central and Western Europe from the GBIF data. (**a**) *Attagenus smirnovi* (**b**) *Attagenus unicolor* (**c**) *Attagenus pellio* and (**d**) *Attagenus brunneus.* Note: increasingly orange shades denote larger numbers.

Among the *Attagenus* spp., *Attagenus smirnovi* has been of much interest in recent years [29,40] and is widely reported across Europe although not so commonly in the Mediterranean area [57,58] (Figure 7a). Almost 1900 European records are found in the GBIF database [57] with more than 10% of these found from Sweden, Finland, Switzerland and Russia. *Attagenus unicolor* appears to have a less northerly distribution (Figure 7b), with more than a third coming from Switzerland and more than 10% from both France and Poland [59]. There are more than 6000 European reports of *Attagenus pellio* in the GBIF database [60], with considerable numbers from Western Europe and the Baltic (Figure 7c). A quarter of the reports are from Sweden, with more than 10% arising from each of France, Switzerland and the UK. *Attagenus brunneus* is less commonly reported with just under 200 European records [61] that show a somewhat southerly distribution (Figure 7d). New *Attagenus* spp. continue to be described e.g., *Attagenus balearicus* from the Balearics, which might represent another island endemic, and *Attagenus vitalii* from France [62].

Austria, in the centre of Europe, might benefit from a geographic position which allows many species to be found, even if they are often at the northern or southern extremes of their range (*Anthrenus museorum*, *Anthrenus fuscus*, *Attagenus smirnovi* or *Attagenus brunneus*).

### 3.5. Pan European Heritage Distribution

The distribution of insects in heritage interiors from various European countries is found in Table 2. The review of insects found indoors by Hasnaoui et al. [63] suggests the presence of *Anthrenus flavipes*, *Anthrenus museorum*, *Anthrenus pimpinellae*, *Anthrenus scrophulariae* and *Anthrenus verbasci* in both heritage sites and residences in France. This aligns with the list from *Insectes du Patrimoine Culturel* in France [25]. *Anthrenus flavipes* caused considerable damage to vertebrate specimens (horn on a skull) held in the Aristotle University of Thessaloniki, Greece [64]. While it may devastate collections in warmer parts of Europe, it is likely to be less significant in cooler northern latitudes, but the presence is poorly mapped (Figure 6h). The list of coleopteran native and alien species causing damage to heritage works from Palermo, Italy [65] includes: *Anthrenus caucasicus*, *Anthrenus flavidus*, *Anthrenus flavipes*, *Anthrenus museorum*, *Anthrenus pimpinellae*, *Anthrenus scrophulariae* and *Anthrenus verbasci*. However, it would seem likely that some of these have northerly distributions, so a presence in Italian interiors would have to be on imported material. Notably *Anthrenus caucasicus*, *Anthrenus flavidus* and *Anthrenus museorum* are not listed in the Italian checklist [66].

In Germany four species are listed [Museums-Schädlinge]: *Anthrenus flavipes* a pest on textiles and insect collections; *Anthrenus museorum* in museums, textile collections and historic houses; *Anthrenus scrophulariae* and *Anthrenus verbasci*, a pest in collections and museums. The What’sEatingYourCollection (WEYC) [27] database for the UK has *Anthrenus verbasci* as the dominant species with more than 2000 records, some two hundred records for *Anthrenus sarnicus*, almost 60 *Anthrenus fuscus*, but only two *Anthrenus flavipes*. *Anthrenus museorum* is very rarely found in buildings in the UK [68], suggesting that *Anthrenus verbasci*, *Anthrenus fuscus* and *Anthrenus sarnicus* are typical of UK museums.

In Switzerland only *Anthrenus verbasci*, *Anthrenus museorum*, *Anthrenus scrophulariae* have been found so far in museums, but here more species can probably be expected, as these observations come only from the German speaking parts of the country (PQ personal observations from traps). *Anthrenus museorum* is tabulated as a common pest in Spain [69]. However, it is not present in the checklist of Spanish Dermestidae [70], although 20 other species are. In Spain no publication specifically lists the species occurring in museums, so the heritage fauna of the Iberian Peninsula remains uncertain. The recently discovered *Anthrenus querneri* from the palace of Schönbrunn in Vienna (SB) has also been claimed from Slovakia and the Czech Republic in 2024 [71].

Misidentification can be a problem as some *Anthrenus* spp. look very similar and need the correct literature, so an intact and good specimen, a good microscope and sometimes expert help is required to identify them correctly. *Anthrenus olgae* and *Anthrenus caucasicus* for example look very similar. The *Anthrenus pimpinellae* group is even more complicated; what was believed to be one species is now a group of perhaps 20 or more species and the male genitalia need to be compared for correct species identification [39].

The review by Hasnaoui et al. [63] suggests the presence of: *Attagenus bifasciatus* Olivier, 1790, *Attagenus brunneus*, *Attagenus cyphonoides* Reitter, 1881, *Attagenus fasciatus* Thunberg, 1795, *Attagenus pellio*, *Attagenus smirnovi* and *Attagenus unicolor* at heritage sites and residences in France. They give more importance to *Attagenus bifasciatus*, another complex [56] and *Attagenus fasciatus*. The list from *Insectes du Patrimoine Culturel* in France [25], gives *Attagenus pellio* and *Attagenus unicolor* in some detail, but *Attagenus bifasciatus*, *Attagenus cyphonoides* and *fasciatus* are also listed albeit briefly. The list of coleopteran native and alien species causing damage to heritage works from Barbara Manachini [65] in Palermo, includes: *Attagenus bifasciatus*, *Attagenus smirnovi*, *Attagenus cyphonoides*, *Attagenus fasciatus*, *Attagenus pellio* and *Attagenus unicolor*. In the UK, the WEYC database gives 700 records for *Attagenus pellio* and a fifth this number as *Attagenus smirnovi*. *Attagenus piceus* Olivier, 1790 is tabled as a common pest in Spain [69], but it does not appear on the check list [68] and may be a synonym *Attagenus unicolor*. Many (42%) *Attagenus* spp. in Spain are unconfirmed [68], and while the well-known museum pests: *Attagenus brunneus*, *Attagenus pellio* and *Attagenus unicolor* are present on the Spanish checklist, the increasingly discussed heritage pest *Attagenus smirnovi* is not confirmed on the list. Identification of the individual *Attagenus* spp. remains difficult e.g., *Attagenus unicolor* and *Attagenus brunneus* are both black, have similar body shape and size, with a curve in the last segment of the antenna is used to differentiate the species [72].

While related *Dermestidae* such as *Trogoderma angustum*, *Trogoderma granarium Everts*, 1898, *Trogoderma glabrum*, *Thylodrius contractus* Motschulsky, 1839 and *Reesa vespulae* are not very common in museums in Austria, differences in Europe can be quite large: *Trogoderma angustum* is for example quite common in northern Germany. Additionally, *Anthrenocerus australis* Hope, 1843 is also found in Germany. All these species may well pose threats to collections similar to *Anthrenus* spp. and *Attagenus* spp., but it is not known which of the *Dermestidae* will benefit most from climate change.

## 4. Conclusions

The study shows that although carpet beetles are common, the catch varies widely across the heritage interiors studied here. In the case of *Anthrenus* spp., they were most abundant in the cooler libraries with *Anthrenus olgae* the most caught, despite its general rarity across many parts of Europe. In Austria, the dermestid community in heritage environments is dominated by *Anthrenus verbasci* and *Anthrenus olgae*. These two species form a core ecological pair, almost always co-occurring so likely share very similar places and dispersal pathways. The less common *Anthrenus museorum*, *Anthrenus scrophulariae* and *Anthrenus fuscus* were more common in the cooler interiors. The *Attagenus* spp. in heritage interiors were dominated by *Attagenus smirnovi* especially in an urban archive and studio (HB) and though *Attagenus pellio* was infrequently found, it was abundant at the unheated Franzensburg Castle (FR). Perhaps, microclimates such as insuated walls, local heat sources or crevices mnght have more amenable climates, or alternatively *Attagenus pellio* may have evolved a cold-adaptation mechanism overwinters.

Further research, more laboratory work on environmental preferences and a better understanding of the European context is required. Not enough is known about the growth range of heritage insects. This could be of relevance to concerns about shifting geographic range under a changing climate. As noted earlier, the contemporary geographic distribution is not always well understood, especially with complex groups e.g., *Anthrenus pimpinellae*. Identification of the species within the heritage environment remains a problem so better awareness and convenient identification keys would improve the understanding of the changing distribution and threat to collections. Doubtless shifts in climate will alter the risk to heritage, so enhanced knowledge is needed to improve the integrated management of museum pests.

## Figures and Tables

**Table 2:** List of species regarded as present in heritage environments in some European countries, is denoted by bold dots. Note: The two letter codes are AT Austria, GE Germany, FR France, IT Italy, UK United Kingdom, SW Switzerland, CZ Czechia, HU Hungary. Cosmopolitan species are marked with a “c” and doubtful claims as “?”.

Species	Authority	AT	GE	FR	IT	UK	SW	CZ	HU
*Anthrenus*									
*caucasicus*	Reitter, 1881	●							
*coloratus*	Reitter, 1881				●				
*flavidus*	Solsky, 1876				●				
*flavipes* c	LeConte, 1854	●	●	●	●	●			
*fuscus*	Olivier, 1789	●	●			●		●	
*museorum* c	Linnaeus, 1761	●	●	●	●	●	●	●	●
*olgae*	Kalik, 1946	●	●			●		●	●
*Pimpinellae* ^1^	Fabricius, 1775		●	●	●				
*querneri*	Holloway, 2024	●							
*sarnicus*	Mroczkowski, 1963	●				●			
*scrophulariae* c	Linnaeus, 1758	●	●	●	●		●	●	●
*verbasci* c	Linnaeus, 1767	●	●	●	●	●	●	●	●
*Attagenus*									
*bifasciatus*	Olivier, 1790			●	●				
*brunneus*	Faldermann, 1835	●				?	?	●	?
*cyphonoides*	Reitter, 1881			●	●				
*fasciatus*	Thunberg, 1795								
*pellio* c	Linnaeus, 1758	●	●			●	●	●	●
*smirnovi*	Zhantiev, 1973	●	●	●	●	●	●	●	●
*unicolor* c	Brahm, 1790	●	●	●	●	●	●	●	●

Note: ^1^ there is no evidence that Anthrenus pimpinellae is cosmopolitan. This belief is perpetuated following decades of misidentification as it appears to be largely restricted to Europe [67].

## Data Availability

The insect trapping data used in this paper is available at: https://zenodo.org/records/18226647 (accessed on 3 March 2026).
